# 365. Hospitalizations Associated with Strongyloidiasis in the United States, 2003-2018

**DOI:** 10.1093/ofid/ofac492.443

**Published:** 2022-12-15

**Authors:** Kengo Inagaki, Richard S Bradbury, Charlotte V Hobbs

**Affiliations:** University of Michigan, Ann Arbor, Michigan; Federation University, Berwick, Victoria, Australia; University of Mississippi Medical Center, Jackson, Mississippi

## Abstract

**Background:**

*Strongyloides stercoralis* is considered to be historically endemic in Appalachia and the American South, but recent surveillance data, especially data evaluating strongyloidiasis associated with hospitalization, are lacking in most parts of the US.

**Methods:**

We performed a population-based retrospective analysis on strongyloidiasis using the *National Inpatient Sample* from 2003-2018. Geographic distribution of strongyloidiasis associated hospitalization was assessed. Logistic regression was used to identify risk factors associated with strongyloidiasis.

**Results:**

We identified 6931 hospitalizations associated with strongyloidiasis during the study period (11.8 per million hospitalizations). The rate of strongyloidiasis was highest in the US Northeast region including the Middle Atlantic division (47.1 cases per million population, adjusted odds ratio, aOR: 2.00 [95% confidence interval, CI: 1.58-2.53]), and the East South Central division (27.5 cases per million population, adjusted odds ratio, aOR: 2.77 [95% confidence interval, CI: 2.02-3.80]) (Figure 1, Figure 2). Older age, male sex, non-white race/ethnicity particularly Hispanic and Asian, non-private insurance, and residence in neighborhoods with low median income were also associated with strongyloidiasis (Table, Figure 2). Immunocompromising conditions, particularly human immunodeficiency virus infection, were present in 41.3% of hospitalizations with strongyloidiasis (Table). In-hospital death was seen in 7.8% of cases with strongyloidiasis-associated hospitalization.

**Figure:**
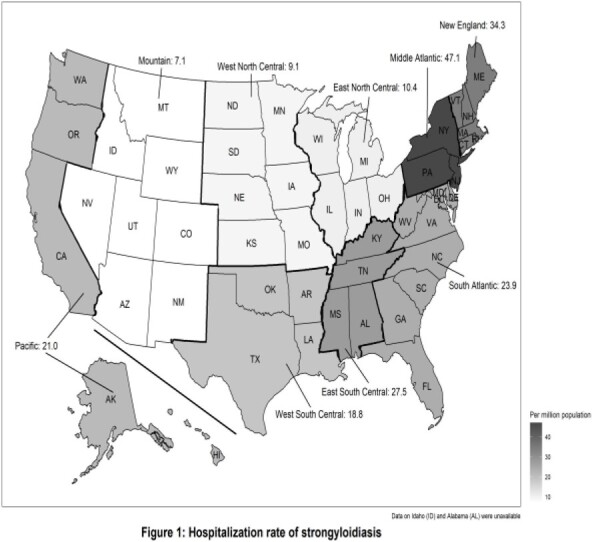

**Figure:**
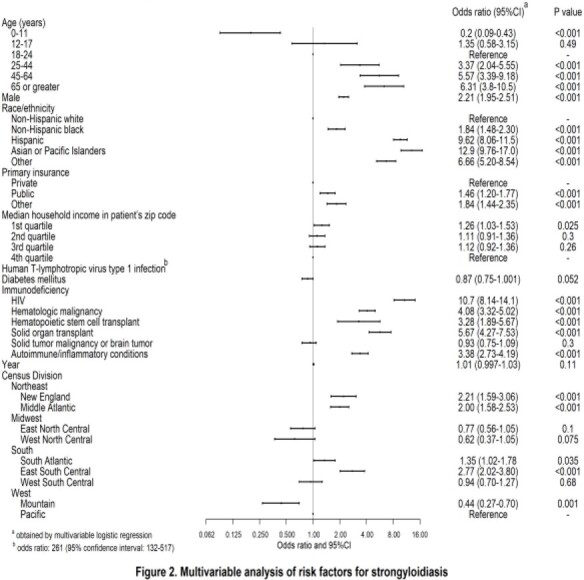

**Figure:**
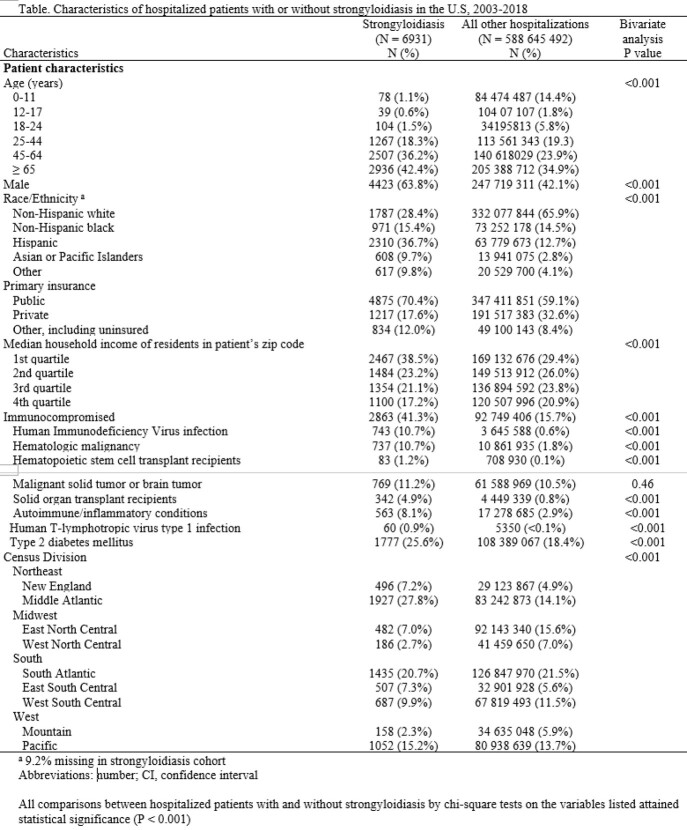

**Conclusion:**

Strongyloidiasis-associated hospitalization is rare in the U.S. but can be associated with mortality. It occurs more frequently in poor and marginalized populations. Immunocompromised conditions were common among hospitalized patients with strongyloidiasis. Enhanced surveillance efforts are needed to inform health policies for improving the health of at-risk populations.

**Disclosures:**

**Charlotte V. Hobbs, MD**, Biofire (Biomerieux): Advisor/Consultant.

